# Evaluation of Scopio Labs X100 Full Field PBS: The first high‐resolution full field viewing of peripheral blood specimens combined with artificial intelligence‐based morphological analysis

**DOI:** 10.1111/ijlh.13681

**Published:** 2021-09-21

**Authors:** Ben‐Zion Katz, Michael D. Feldman, Minychel Tessema, Dan Benisty, Grace Stewart Toles, Alicia Andre, Bronka Shtreker, Fatima Maria Paz, Joshua Edwards, Darrin Jengehino, Adam Bagg, Irit Avivi, Olga Pozdnyakova

**Affiliations:** ^1^ Division of Hematology Tel Aviv Sourasky Medical Center Tel Aviv Israel; ^2^ Sackler Faculty of Medicine Tel Aviv University Tel Aviv Israel; ^3^ Department of Pathology and Laboratory Medicine University of Pennsylvania Philadelphia PA USA; ^4^ Department of Pathology, Brigham and Women's Hospital Boston MA USA

**Keywords:** Artificial Inteligence, blood smear, laboratory automation, morphology

## Abstract

**Background:**

Current digital cell imaging systems perform peripheral blood smear (PBS) analysis in limited regions of the PBS and require the support of manual microscopy without achieving full digital microscopy. We report a multicenter study that validated the Scopio Labs X100 Full Field PBS, a novel digital imaging system that utilizes a full field view approach for cell recognition and classification, in a decision support system mode.

**Methods:**

We analyzed 335 normal and 310 abnormal PBS from patients with various clinical conditions and compared the performance of Scopio's Full Field PBS as the test method, with manual PBS analysis as the reference method. Deming regression analysis was utilized for comparisons of WBC and platelet estimates. Measurements of WBC and platelet estimation accuracy along with the agreement on RBC morphology evaluation were performed. Reproducibility and repeatability (R&R) of the system were also evaluated.

**Results:**

Scopio's Full Field PBS WBC accuracy was evaluated with an efficiency of 96.29%, sensitivity of 87.86%, and specificity of 97.62%. The agreement between the test and reference method for RBC morphology reached 99.77%, and the accuracy for platelet estimation resulted in an efficiency of 94.89%, sensitivity of 90.00%, and specificity of 96.28%, with successful R&R tests. The system enabled a comprehensive review of full field PBS as shown in representative samples.

**Conclusions:**

Scopio's Full Field PBS showed a high degree of correlation of all tested parameters with manual microscopy. The novel full field view of specimens facilitates the long‐expected disengagement between the digital application and the manual microscope.

## 1 INTRODUCTION

Complete blood count (CBC) with peripheral blood smear (PBS) is a rapid common test that serves as a screening tool offering insights into patients’ clinical conditions and guiding further laboratory workup. Despite the good performance of CBC analyzers, their limited capacity to identify morphological variations and abnormalities of blood cellular components^1^, ^2^, ^3^ led to the establishment of a set of rules to trigger manual blood smear review following the outcome of CBC tests that is specific for each hematology laboratory.^4^, ^5^

The need to improve and standardize white blood cell (WBC), red blood cell (RBC), and platelet recognition has led to the development of several digital cell imaging systems that utilize various algorithms and methods to automate PBS image analysis, including image segmentation, feature extraction and selection, and pattern classification.^6^ To date, a single vendor predominates morphological digital analyzers in hematology laboratories worldwide.^6^, ^7^ While current digital image analyzers are constantly improving and expanding, the main drawback of this field is that only limited fields of view (FOV) from the PBS are available for review.^6^, ^7^ As a mitigation, many of the digitally analyzed samples are also manually reviewed under a microscope, specifically those that contain cellular abnormalities, as detailed in the ICSH recommendations.^6^ Hence, the sometimes‐redundant triangle of the PBS, the digital image analyzer, and the manual microscope cannot easily be breached utilizing current technologies.

Our multicenter study evaluated and validated the FDA cleared Scopio Labs X100 Full Field PBS system (Scopio's Full Field PBS), a novel digital PBS morphological analyzer with full field specimens view as described in Supplementary 1. We analyzed 645 peripheral blood specimens, of which 335 were normal CBC and 310 were abnormal CBC collected from patients with various infectious or neoplastic conditions and compared the WBC differential, RBC morphology evaluation, and platelet estimation performance by the Scopio's Full Field PBS, with traditional manual PBS analysis performed by experienced medical technologists according to the Clinical and Laboratory Standards Institute H20, 2nd addition (H20‐A2).^8^ Here, we report a high degree of correlation between the two methods among the WBC classes, RBC morphology evaluation, and platelet estimation, as well as repeatability and reproducibility. In addition, we include representative full field PBS scans for evaluation of the capacity of this novel approach.

## 2 MATERIAL AND METHODS

### 2.1 Scopio labs X100 system

Scopio Labs X100 system is manufactured by Scopio Labs, Tel Aviv, Israel. The system is operated by a browser‐based application, namely, the application does not require specific software installed, and may be accessed from any workstation running a browser, inside the secure medical facility network or securely connected to it remotely. The system is based on a computational photography approach, where a series of low‐resolution full field images of the specimen are acquired by low power/wide field objective, and reconstructed into a high‐resolution full field image based on a physical model (Supplementary 1A). The system includes automated platelet location and pre‐estimation, and WBC pre‐classification by artificial intelligence (AI) based tools into the following three groups: 1) main WBC classes—neutrophils (including segmented and bands forms), lymphocytes, monocytes, eosinophils, basophils; 2) other WBC classes—immature myeloid cells (promyelocytes, metamyelocytes and myelocytes), blast cells, lymphocyte variant forms, plasma cells; 3) non‐WBC classes: nucleated red blood cells and smudge cells. Both WBC and platelet pre‐classifications operate as a decision support system (DSS), requiring the operator to review the pre‐classified data generated by the system, approve, or correct it. So far, DSS is the only mode cleared by the FDA for such analyzers.

### 2.2 Patients and samples

Three clinical sites participated in the multicenter study. These sites included Brigham and Women's Hospital (BWH), designated site #1; the Hospital of the University of Pennsylvania (HUP), designated site #2; and Tel‐Aviv Sourasky Medical Center (TASMC), designated site #3. The study was approved by the corresponding local Institutional Review Boards according to the declaration of the Helsinki accord. The study design was based on the CLSI H20‐A2^8^ guidelines.

Specimens were collected and analyzed according to various conditions detailed in Table 1, resulting in a total sample size of 645 specimens. Six slides were prepared from each specimen. The clinically abnormal specimens were collected from patients according to distinct clinical categories, as specified in the CLSI H20‐A2.^8^ The distribution of samples across sites is shown in Table 1, and the demographics of the patients sampled are summarized in Supplementary 2.

**TABLE 1:** Peripheral blood smears’ distribution and classification across three testing sites and representative samples

Clinical condition	Multi center	HUP	TASMC	BWH	Representative sample links
Total	645	224	219	202	‐
Normal	335	116	115	104	Normal
Abnormal	310	108	104	98	‐
Distribution of abnormal samples					
Acute inflammation/Bacterial infection	49	13	19	17	Acute inflammation/Bacterial infection
Chronic inflammation	32	13	8	11	Chronic inflammation
Parasitic infection/Allergic reaction	44	18	12	14	Parasitic infection/Allergic reaction
Viral infection	41	13	14	14	Viral infection
Aplastic anemia/Chemotherapy	29	13	10	6	Aplastic anemia/Chemotherap
Lymphopenia	42	13	14	15	Lymphopenia
Acute leukemia	33	12	10	11	Acute leukemia
Severe anemia/Myeloproliferative disorders	40	13	17	10	Severe anemia/Myeloproliferative disorders

Note

Peripheral blood smears’ distribution and classification across three testing sites, and representative samples. HUP, the Hospital of the University of Pennsylvania Hospital; TASMC, Tel‐Aviv Sourasky Medical Center; BWH, Brigham and Women's Hospital. Right column: links for representative samples for each clinical category.

### 2.3 Sample preparation

The method of sample preparation depended on site protocols and differed slightly for each site. At site #1, specimens were collected into a spray‐coated K2 EDTA 3.6 mL vacuum tube (BD Vacutainer). PBS were prepared, within four hours and at room temperature, by the Sysmex SP‐10 which is a fully automated hematology slide preparation and staining system, on glass slides (Micro Slides MS‐101; Pre‐cleaned, Frosted, Grounded edges. Sysmex America, Inc).

At site #2, specimens were collected into a spray‐coated K2 EDTA 3.6 mL vacuum tube (BD Vacutainer). PBS were prepared, within four hours and at room temperature, by the Beckman Coulter Unicell DxH Slidemaker Stainer (Brea, CA, USA), on glass slides (DxH Slides, Beckman Coulter).

At site #3, specimens were collected into a spray‐dried K3 EDTA 3.6 mL vacuum tube (Greiner). PBS were prepared, within four hours and at room temperature, by Beckman Coulter Slide Maker Stainer, on glass slides (SP‐Slides, Sysmex). Specific staining protocols and sample preparations are detailed in Supplementary 3.

### 2.4 PBS analysis area

Scopio's Full Field PBS locates an optimal analysis area for each PBS to include the monolayer area as well as the feathered edge (Supplementary 1D). On average, the monolayer part of the scan is 0.38 cm^2^, equivalent to 1000 high power fields (100X magnification). The adaptive scan feature contributed to a robust morphological analysis of short and long smears. No restrictions were applied on the analysis area for the manual review.

### 2.5 PBS evaluation

Scopio's Full Field PBS performs WBC analysis by an artificial intelligence‐based classifier, in a decision support system (DSS) mode. A total of 645 specimens were analyzed for WBC differentials, RBC morphology and platelets estimation by two independent operators, at three sites, using Scopio's Full Field PBS (test arm) and manual microscopy (reference arm). All six operators that participated in the study were qualified and certified to perform PBS morphological analysis by their respective site requirements. In addition, each site had an arbitrator in case of disagreement between the two operators in the reference arm only.

200‐WBC differentials were evaluated as described in the statistical analysis section. If less than 200 WBC were available for analysis in one PBS (eg, in the cases of leukopenia), additional slides from the same specimen were analyzed. As a DSS, Scopio's Full Field PBS results for the WBC differentials were approved (or modified where required) by the operators (Supplementary 1). In the reference arm, the operators performed a manual WBC differential using a manual microscope.

The platelet estimation was derived by automatically locating and counting platelets in 10 FOVs (Supplementary 1E), and multiplying the total count by a constant factor specific to each center and method. As a DSS, platelet detections were approved (or modified where required) by the operators. For the reference method, the operators manually counted platelets in 10 FOVs and calculated a platelet estimation.

Twenty‐two parameters of RBC morphology were evaluated by the operators (Supplementary 4). In the test arm, the operators reviewed the digitally scanned PBS with an overlaid grid, with each grid cell proportionally representing a single high‐powered manual microscope FOV. For the reference method, the operators manually evaluated the RBC morphology.

### 2.6 Repeatability

Following CLSI’s EP05‐A3 Evaluation of Precision of Quantitative Measurement Procedures, 3rd Edition (CLSI’s EP05‐A3),^9^ standardized “20 × 2 × 2” (20 days, 2 runs, 2 replicas) repeatability experiment was conducted for 15 test samples (8 normal, 7 abnormal) which were randomly selected from within each clinical group. For each test sample, WBC pre‐classification and platelet estimation results were analyzed with a two‐way nested ANOVA, and standard deviation (SD) estimates with 95% confidence interval (CI) for the repeatability, between‐run (within‐day), between‐day, and within‐laboratory variance components were calculated.

### 2.7 Reproducibility

Following CLSI’s EP05‐A3,^9^ a standardized “3 × 5 × 5” (3 devices, 5 days, 5 replicas) reproducibility experiment was conducted for 10 test samples (5 normal and 5 abnormal), which were randomly selected from within each clinical group. For each test sample, WBC pre‐classification and platelet estimation results were analyzed with a two‐way nested ANOVA, and SD estimates with 95% CI for the between‐day (within‐site), between‐site, within‐laboratory variance, and reproducibility components were calculated.

### 2.8 Statistical analyses

To account for operator‐related differences between the test and reference methods, the CLSI H20‐A2 technique of averaging the two operators’ measurements in the reference arm and the test arm was employed, with a complementary bootstrap method^10^ using operator's individual data (ie, without averaging two operators’ measurements). These two statistical techniques, that yielded comparable results, were used with the Deming regression analysis for WBC and platelet estimates, calculations of efficiency (agreement), sensitivity and specificity of WBC abnormality grading and platelet estimations.

Deming regression analysis was performed on WBC differential results for neutrophils, lymphocytes, monocytes, eosinophils, and platelet estimates, with a complementary Bland‐Altman^11^ analysis for method bias.

Following the CLSI H20‐A2 guidance, for accuracy measurements in terms of efficiency (agreement), sensitivity and specificity of WBC abnormality grading, a distributional WBC group and a morphological WBC group were defined. The distributional WBC groups and the morphological WBC groups are detailed in Table 2. In both reference and test methods, WBC cell types with a count outside their defined normal ranges were marked as abnormal. Sequentially, each result from the test arm was given a label of true negative (TN), when no abnormality was detected in neither the test nor reference arm, true positive (TP) when an abnormality was detected in both the test arm and reference arm, false negative (FN) when no abnormality was detected in the test arm, but was detected in the reference arm, or false positive (FP), when an abnormality was detected in the test arm, but not in the reference arm. The TP, TN, FP, and FN values were summarized for all specimens, by groups, and efficiency, sensitivity, and specificity were calculated.

**TABLE 2:** Comparison of distributional normal ranges (%) and morphological normal ranges (%) between the manual and digital methods across three sites

Distributional normal ranges						
Cell type	HUP		TASMC		BWH	
	Manual microscope	Full Field microscope	Manual microscope	Full Field microscope	Manual microscope	Full Field microscope
Neutrophils	47.50‐84.50	45.33‐84.50	46.00‐76.50	42.00‐75.00	53.06‐81.00	51.50‐79.94
Lymphocytes	8.50‐46.23	7.00‐45.82	10.00‐39.50	11.00‐37.00	8.50‐29.26	7.00‐30.07
Monocytes	1.00‐10.00	0.90‐11.00	2.00‐12.00	1.41‐11.50	2.06‐8.94	1.06‐8.50
Eosinophils	0.00‐5.50	0.00‐5.50	0.00‐8.00	0.00‐6.50	0.00‐4.00	0.00‐4.44
Basophils	0.00‐2.00	0.00‐2.01	0.00‐2.00	0.00‐2.50	0.00‐1.52	0.00‐1.97
Morphological normal ranges						
NRBC	0.00‐0.50	0.00‐1.00	0.00‐0.00	0.00‐0.50	0.00‐1.00	0.00‐0.50
Blasts	0.00‐0.50	0.00‐0.50	0.00‐0.00	0.00‐0.00	0.00‐0.00	0.00‐0.00
Variant lymphocytes	0.00‐5.50	0.00‐9.50	0.00‐16.00	0.00‐19.00	0.00‐11.94	0.00‐14.00
Immature granulocytes	0.00‐1.00	0.00‐2.00	0.00‐0.50	0.00‐0.50	0.00‐0.50	0.00‐1.00
Plasma cells	0.00‐0.00	0.00‐0.51	0.00‐0.00	0.00‐0.29	0.00‐0.00	0.00‐0.00

Note

Comparison of distributional normal ranges (%) and morphological normal ranges (%) between the manual and digital methods across three sites. Immature myeloid cells include metamyelocytes, myelocytes, and promyelocytes. Variant lymphoid cells include atypical lymphocytes, aberrant lymphocytes, and large granular lymphocytes. HUP, Hospital of the University of Pennsylvania Hospital; TASMC, Tel‐Aviv Sourasky Medical Center; BWH, Brigham and Women's Hospital.

Platelet estimation accuracy measurements for efficiency (agreement), sensitivity, and specificity calculations were conducted similar to the WBC groups.

For the RBC analysis, 22 RBC characteristics were subdivided into 5 groups: color (chromasia), shape, size, inclusions, and arrangement (Supplementary 4). A four‐by‐four contingency table for each RBC group was calculated by summing all the grades given (0, +1, +2, +3) in the two methods, including results from both operators, and overall agreement between grades given in both methods was measured.^12^

## 3 RESULTS

**FIGURE 1:**
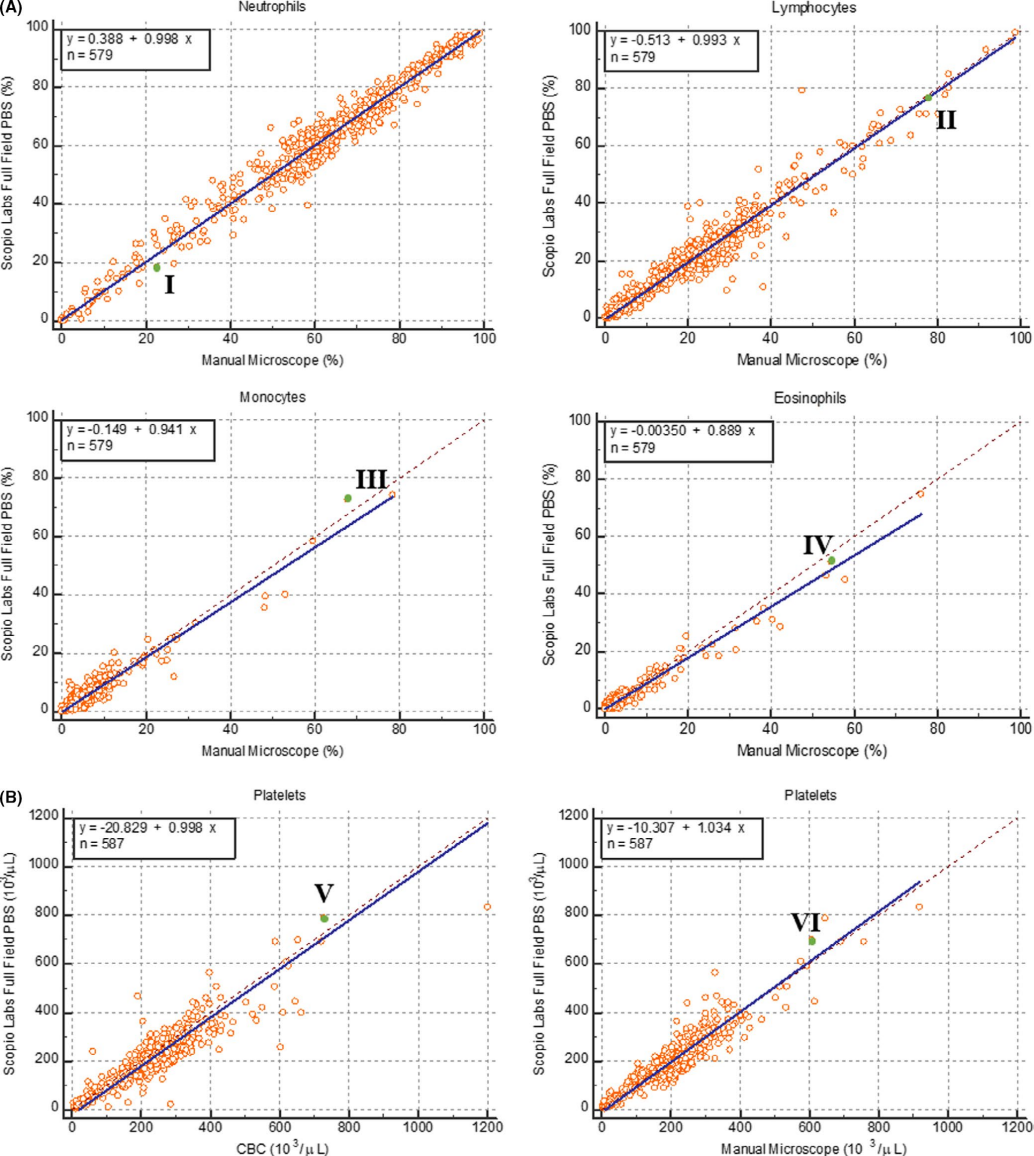
A, Comparison between manual differential count and Scopio Labs Full Field PBS system for neutrophils, lymphocytes, monocytes, and eosinophils. Correlation coefficients (R^2^) are indicated for each cell types. Representative samples from the correlation curves (green dots) are indicated in Roman letters and can be viewed in the links indicated herein: I – Neutrophils: https://demo.scopiolabs.com/#/view_scan/65e8a4bd‐4fa2‐4447‐a6dc‐ad0cf977df22; II – Lymphocytes: https://demo.scopiolabs.com/#/view_scan/507d01a1‐f4a1‐4ece‐b117‐ef663f6fdea3; III – Monocytes: https://demo.scopiolabs.com/#/view_scan/dfd17545‐e517‐43e1‐8957‐f03e40ab07e5; IV – Eosinophils: https://demo.scopiolabs.com/#/view_scan/65da1bcf‐521f‐46ef‐83e7‐ea7b20854e01. B, Comparison between CBC‐derived platelet count (left) and manual microscopy platelet estimation (right) to Scopio Labs Full Field PBS system platelet estimation. Correlation coefficients (R^2^) are indicated for each comparison. Representative samples from the correlation curves (green dots) are indicated in Roman letters and can be viewed in the links indicated herein: V – CBC comparison: https://demo.scopiolabs.com/#/view_scan/d047ca7f‐728a‐4435‐807c‐b782aea96cb9; VI – manual microscopy estimation comparison: https://demo.scopiolabs.com/#/view_scan/47ced5c4‐cda2‐4837‐aa99‐7f296fa4cd37

The correlation coefficients for neutrophils, lymphocytes, monocytes and eosinophils, were 0.98, 0.96. 0.95, and 0.98, respectively; the slope values of 1, 0.99, 0.94, and 0.89, respectively, and the intercept values of 0.39, −0.51. −0.15, and −0.004, respectively (Figure 1A). A low number of basophils (up to 5%) did not allow for a meaningful statistical analysis (data not shown). Additionally, a Bland‐Altman analysis was performed on the WBC differentials and demonstrated no test method bias (Supplementary 5). Normal WBC reference ranges were calculated based on 335 normal CBC samples (Table 1). The measurements of the major WBCs normal ranges are shown in Table 2. The normal ranges were comparable between the two methods and between the three sites. The accuracy of the morphological abnormalities, distributional abnormalities, and total accuracy were 96.82%, 95.75%, and 96.29% (with 95% confidence intervals of 96.12% to 97.43%, 94.95% to 96.46%, and 95.77% to 96.76%), respectively. The sensitivity of the same groups was 85.46%, 88.83%, and 87.86% (with intervals of 80.19% to 89.78%, 85.94% to 91.31%, and 85.38% to 90.06%), respectively. The specificity of the same groups was 97.79%, 97.43%, and 97.62% (with intervals of 97.16% to 98.31%, 96.70% to 98.03%, and 97.16% to 98.02%), respectively. The results above are after the corrections/approvals by the operators in the DSS mode. The performance of the pre‐classified outcome and its improvement by the operators (the DSS stage) are detailed in Supplementary 6.

RBC morphology evaluation results are shown in Table 3. The agreement of the RBC groups (color, shape, size, inclusions, arrangement and overall) was 99.49%, 99.77%, 99.61%, 100.00%, 96.65%, and 99.77% (with 95% confidence intervals of 99.14% to 99.73%, 99.68% to 99.84%, 99.36% to 99.78%, 99.93% to 100.00%, 95.52% to 97.57% and 99.71% to 99.83%), respectively, with no statistical significance between the sites.

**TABLE 3:** Comparison between manual RBC analysis of microscopy (reference) and Scopio Labs Full Field PBS (test)

RBC morphology group	Multi‐center	HUP	TASMC	BWH
Color	99.49% 99.14%‐99.73%	98.88% 97.96%‐99.46%	100.00% 99.57%‐100.00%	99.63% 98.92%‐99.92%
Shape	99.77% 99.68%‐99.84%	99.94% 99.84%‐99.99%	99.96% 99.86%‐100.00%	99.36% 99.09%‐99.57%
Size	99.61% 99.36%‐99.78%	99.11% 98.45%‐99.54%	99.92% 99.57%‐100.00%	99.83% 99.41%‐99.98%
Inclusions	100.00% 99.93%‐100.00%	100.00% 99.79%‐100.00%	100.00% 99.79%‐100.00%	100.00% 99.77%‐100.00%
Arrangement	96.65% 95.52%‐97.57%	99.11% 97.73%‐99.76%	90.97% 87.87%‐93.50%	100.00% 99.09%‐100.00%
Overall	99.77% 99.71%‐99.83%	99.75% 99.63%‐99.84%	99.97% 99.91%‐99.99%	99.59% 99.44%‐99.72%

Note

Comparison between manual RBC analysis of microscopy (reference) and Scopio Labs Full Field PBS (test). The range and average agreement between reference and test methods in 5 morphological groups across the three sites are presented (%). HUP, Hospital of the University of Pennsylvania Hospital; TASMC, Tel‐Aviv Sourasky Medical Center; BWH, Brigham and Women's Hospital.

For platelets estimations, the slope, correlation, and intercept between the test and reference methods were 1.03, 0.94, and −10.31, respectively, and between the test method and CBC were 0.998, 0.91, and −20.83, respectively (Figure 1B). The comparison between the test and reference method resulted in an accuracy of 94.89%, sensitivity of 90.00%, and specificity of 96.28% (with 95% confidence intervals of 92.78% to 96.53%, 83.51% to 94.57%, and 94.11% to 97.82%, respectively). A Bland‐Altman analysis was performed on the platelet estimations and demonstrated no bias between the test and reference methods (Supplementary 7).

**FIGURE 2:**
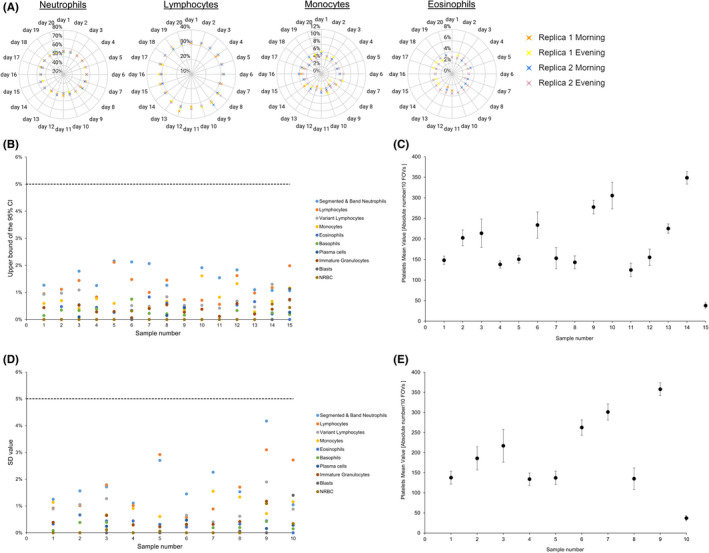
A, A representative layout of the repeatability measurement of a normal sample for neutrophils, lymphocytes, monocytes, and eosinophils. Two replicas of the samples for each cell type are presented along the 20‐day period of the experiment. The values are in % of the differential. B, Repeatability results of the different WBC types. Upper bound of the SD’s 95% CI values for within‐laboratory precision component is presented for the different WBC types in the 15 tested samples. Each cell type is presented according to the color code (right). All values were below the predefined acceptance criteria of 5% (dashed line). C, Repeatability results for platelets estimation. The mean platelet values for each of the 15 tested sample are presented. The vertical error segments representing SD within‐laboratory precision component. All SD values were below the predefined acceptance criteria of 50 platelets. D, Reproducibility results of the different WBC types. The SD values of the reproducibility component for the different WBC types in the 10 tested samples are shown. Each cell type is presented according to the color code (right). All SD values were below the predefined acceptance criteria of 5% (dashed line). E, Reproducibility results for platelets estimation. The mean platelets value for each of the 10 tested sample is presented. The vertical error segments representing SD values of the reproducibility component

The repeatability and reproducibility study supported high levels of repeatability and reproducibility regarding both WBC and platelets measurements of the Scopio's Full Field PBS. A representative layout for the repeatability measurement of a normal sample is shown in Figure 2A. Standard deviation (SD) and 95% confidence intervals (CI) estimates were constructed for each WBC subclass. Upper bound of the SD’s 95% CI values for within‐laboratory precision component were plotted for the different WBC types in the tested samples. All values were below the pre‐defined acceptance criteria of 5% (Figure 2B). For platelets, Figure 2C plots the mean platelet values for each tested sample, with the vertical error segments representing SD within‐laboratory precision component. All SD values were below the pre‐defined acceptance criteria of 50 platelets. The WBC reproducibility results are shown in Figure 2D, with SD values of the reproducibility component, for the different WBC types in the tested samples. All SD values were below the pre‐defined acceptance criteria of 5% (Figure 2D). For platelets, the results are shown in Figure 2E, with the mean platelets value for each tested sample plotted with the vertical error segments representing SD values of the reproducibility component. All SD values were below the pre‐defined acceptance criteria of 50 platelets. In order to assess the capacity of the system to identify platelets clumps, we analyzed ten samples with confirmed pseudothrombocytopenia. We detected platelets clumps in 10/10 of the samples, including two samples with clumps located at the feathered edge of the smear. Representative samples are shown in Supplementary 8.

The revolutionary full field capability of the system, that includes the PBS monolayer and feathered edge, enables experts to gain general slide context, which is critical for proper clinical decision‐making. We included several full field PBS scans of several clinical examples (Figure 1, representative samples; Table 1, representative samples links; Supplementary 8).

## 4 DISCUSSION

The goal of our study was to assess and validate the Scopio Labs X100 Full Field PBS application. In addition to demonstrating a high degree of correlation with the manual method for WBC classification, RBC morphology evaluation, and platelet estimation, Scopio's Full Field breakthrough technology enables viewing of whole blood smears *via* a modern browser‐based application, accompanied by pre‐classification of WBC and platelets estimation (links in Table 1 and Figure 1). While the performance of existing digital microscopic systems in classification of WBC is generally adequate in a decision support system (DSS) mode, the option of full field specimen viewing during PBS analysis is an unmet need.^6^, ^7^ The correct RBC morphological classification is problematic, and the automated identification of highly informative RBC forms such as schistocytes or teardrop cells need to be significantly reviewed.^6^

As noted by the ICSH recommendations, abnormal leukocytes may be under‐represented in limited‐area digital smear analyses of flagged‐CBC samples.^6^ Scopio's Full Field PBS enables full screening of the PBS, allowing the correct identification of the problematic cases, as demonstrated in the links provided in the manuscript. For example, in the case with viral infection, reactive lymphocytes as well as numerous apoptotic lymphocytes are clearly observed. In the case of a patient on chemotherapy, abnormalities of RBC are present, including microcytosis, hypochromia and occasional teardrop cells and schistocytes, apart from the profound leukopenia and thrombocytopenia. In the specimen with acute leukemia, anisocytosis, frequent teardrop cells, and ovalocytes are evident, the appearance of the blasts is myelomonocytic accompanied by immature/aberrant monocytes, and aberrant giant platelets are found, suggesting that the acute myeloid leukemia may have evolved from a myeloproliferative neoplasm or chronic myelomonocytic leukemia. The degree of RBC abnormalities, such as the prevalence of schistocytes or malarial trophozoites can be evaluated from a full field view of a PBS (Supplementary 8A, B). In the case of malaria, most infected RBCs include one or two parasites, with rare RBCs containing three or more parasites (Supplementary 8B). In addition, RBC arrangement abnormalities such as rouleaux formation (Supplementary 8C) are clearly observed in the full field view of the PBS.

An artifact sometimes observed in CBCs is pseudothrombocytopenia,^5^, ^14^ with platelet clumping as a common cause. However, the clumps are frequently unevenly distributed in a PBS, leaving large sections devoid of platelets (Supplementary 8D, E). Furthermore, large clumps may accumulate at the feathered edge of the PBS, and out of the range for digital analyzers. Viewing of only a small segment of the PBS, either manually or by an automated morphological analyzer may cause misinterpretation of such cases leading to potentially inappropriate treatment decisions.^4^, ^5^ Previous automated digital microscopes fail to identify many of the specimens containing platelet clumps due to their narrow field of view, rendering manual microscope reviewing of samples with CBC‐derived thrombocytopenia essential.^6^, ^7^, ^13^, ^14^, ^15^ Previous digital microscopy users report limitations with respect to both platelets and RBC analysis,^14^ and manual PBS analysis is recommended in various conditions.^15^ As we show here (Supplementary 8), Scopio's Full Field approach waives the need to default back to a manual microscopy.

The ICSH recommendations raised concerns about possible differences between digital cellular images and the manual observation practice of laboratory specialists.^6^ However, in a full field digital setting, our study points to a good comparison between methodologies, both with respect to the classification of WBC types in a DSS mode, and to the recognition of aberrant cell types. With respect to platelet estimation, Scopio's Full Field PBS automatically identifies and produces platelet concentration estimates in a DSS mode, with performance similar to both CBC analyzers and to manual platelet estimates, and no significant bias. These performances were demonstrated here in a multi‐center study involving multiple qualified operators, utilizing various slide‐makers and staining protocols. Notably, case review times were documented throughout the study, in both the reference arm (manual microscope) and test arm (Scopio's Full Field PBS). Under clinical study settings, where each examiner reported on a 200‐WBC differential, complete RBC morphology evaluation and platelet estimation based on 10 FOVs, the median time for manual review was 20:00 minutes per case, and the median time for Scopio's Full Field PBS review was 7:46 minutes, a 60% improvement of workflow efficiency.

Scopio Labs X100 Full Field PBS adaptive scanning area of the slide, meant to locate the feathered edge and the monolayer in short and long smears is fully automated, without the user being able to override it. While the automatic scanned area was suitable throughout the study, enabling override option seemed reasonable. On average, scan and pre‐classification times were 4 minutes per slide, but were up to 7 minutes for long smears. Also, the AI‐based tools were available for the pre‐classification of 16 WBC classes and platelets detection only. RBC morphology evaluation remains a completely manual workflow but is based on more than 1000FOVs images of the full field. Pre‐classification of the leukocytes did not include all types of aberrant cells (e.g., various types of lymphoma cells). User manual reclassification of the WBCs, specifically in the aberrant cases, was required to achieve higher level of sensitivity (7% average increase, Supplementary 6). For platelet estimations, reclassification did not yield notable improvement in accuracy.

Scopio Labs Full Field PBS application may help to bridge the gap between the manual and digital microscopic PBS review, especially in cases where the context of the slide is important in making a diagnosis, and in settings when a member of the clinician and diagnostic team is not present on site and/or at a laboratory performing the test. Additionally, the browser‐based solution presented herein can be integrated into external quality assessment (EQA) schemes, and to wide scale remote training programs, to simulate real‐life blood smear comprehensive morphological analysis. There are thus numerous avenues in which this technology may be employed for the benefit of hematology‐based health care.

## CONFLICT OF INTEREST

Ben‐Zion Katz is a consultant to Scopio Labs.

## AUTHORS CONTRIBUTIONS

B‐Z K, I A, M D F, and O P involved in conceptualization; B‐Z K, A B, and O P involved in methodology; M T, D B, G S T, A A, B S, F M P, J E, and D J analyzed and investigated the study; B‐Z K and O P wrote the original draft; B‐Z K, M D F, A B, I A, and O P wrote, reviewed, and edited the article; I A and M D F supervised the study. All authors read and approved the final manuscript.

## Supporting information

Supplement S1

Supplement S2

Supplement S3

Supplement S4

Supplement S5

Supplement S6

Supplement S7

Supplement S8

## Data Availability

The data that support the findings of this study are available from the corresponding author upon reasonable request.

## References

[1] Döhner H , Estey E , Grimwade D , et al. Diagnosis and management of AML in adults: 2017 ELN recommendations from an international expert panel. Blood. 2017;129:424‐447. 27895058 10.1182/blood-2016-08-733196 PMC5291965

[2] Cerny J , Rosmarin AG . Why does my patient have leukocytosis? Hematol Oncol Clin North Am. 2012;26:303‐viii. 22463829 10.1016/j.hoc.2012.01.001

[3] Saha M , McDaniel JK , Zheng XL . Thrombotic thrombocytopenic purpura: pathogenesis, diagnosis and potential novel therapeutics. J Thromb Haemost. 2017;15:1889‐1900. 28662310 10.1111/jth.13764 PMC5630501

[4] Barnes PW , McFadden SL , Machin SJ , Simson E . international consensus group for hematology. The international consensus group for hematology review: suggested criteria for action following automated CBC and WBC differential analysis. Lab Hematol. 2005;11:83‐90. 16024331

[5] Gulati G , Song J , Florea AD , Gong J . Purpose and criteria for blood smear scan, blood smear examination, and blood smear review. Ann Lab Med. 2013;33:1‐7. 23301216 10.3343/alm.2013.33.1.1 PMC3535191

[6] Kratz A , Lee SH , Zini G , Riedl JA , Hur M . Machin S Digital morphology analyzers in hematology: ICSH review and recommendations. Int J Lab Hematol. 2019;41:437‐447. 31046197 10.1111/ijlh.13042

[7] Billard M , Lainey E , Armoogum P , Alberti C , Fenneteau O , Da Costa L . Evaluation of the CellaVision DM automated microscope in pediatrics. Int J Lab Hematol. 2010;32:530‐538. 20132350 10.1111/j.1751-553X.2009.01219.x

[8] Reference Leukocyte (WBC) Differential Count (Proportional) and Evaluation of Instrumental Methods; Approved Standard—Second Edition. CLSI document H20–A2. Wayne, PA: Clinical and Laboratory Standard Institute; 2007.

[9] CLSI , Evaluation of Precision of Quantitative Measurement Procedures; Approved Guideline – Third Edition. CSLI document EP05‐A3. Wayne, PA: Clinical and Laboratory Standard Institute; 2014.

[10] Davison AC , Hinkley D . Bootstrap Methods and their Application. :Cambridge University Press; 1997.

[11] Bland JM , Altman DG . Measuring agreement in method comparison studies. Stat Methods Med Res. 1999;8:135‐160. 10501650 10.1177/096228029900800204

[12] Palmer L , Briggs C , McFadden S , et al. ICSH recommendations for the standardization of nomenclature and grading of peripheral blood cell morphological features. Int J Lab Hematol. 2015;37:287‐303. 25728865 10.1111/ijlh.12327

[13] Gulati G , Uppal G , Florea AD , Gong J . Detection of platelet clumps on peripheral blood smears by CellaVision DM96 system and microscopic review. Lab Med. 2014;45:368‐371. 25316670 10.1309/LM604RQVKVLRFXOR

[14] VanVranken SJ , Patterson ES , Rudmann SV , Waller KV . A survey study of benefits and limitations of using CellaVision DM96 for peripheral blood differentials. Clin Lab Sci. 2014;27:32‐39. 24669444

[15] Da Costa L . Digital image analysis of blood cells. Clin Lab Med. 2015;35:105‐122. 25676375 10.1016/j.cll.2014.10.005

