# Supplementary material for: Evaluation of Scopio Labs X100 Full Field PBS: The first high‐resolution full field viewing of peripheral blood specimens combined with artificial intelligence‐based morphological analysis
Source: Int J Lab Hematol. 2021 Sep 21;43(6):1408–16. doi: 10.1111/ijlh.13681 (PMC9293172; doi:10.1111/ijlh.13681)
Supplement: Supplementary file 1 — Supplement S1 [file IJLH-43-1408-s001.docx]

**
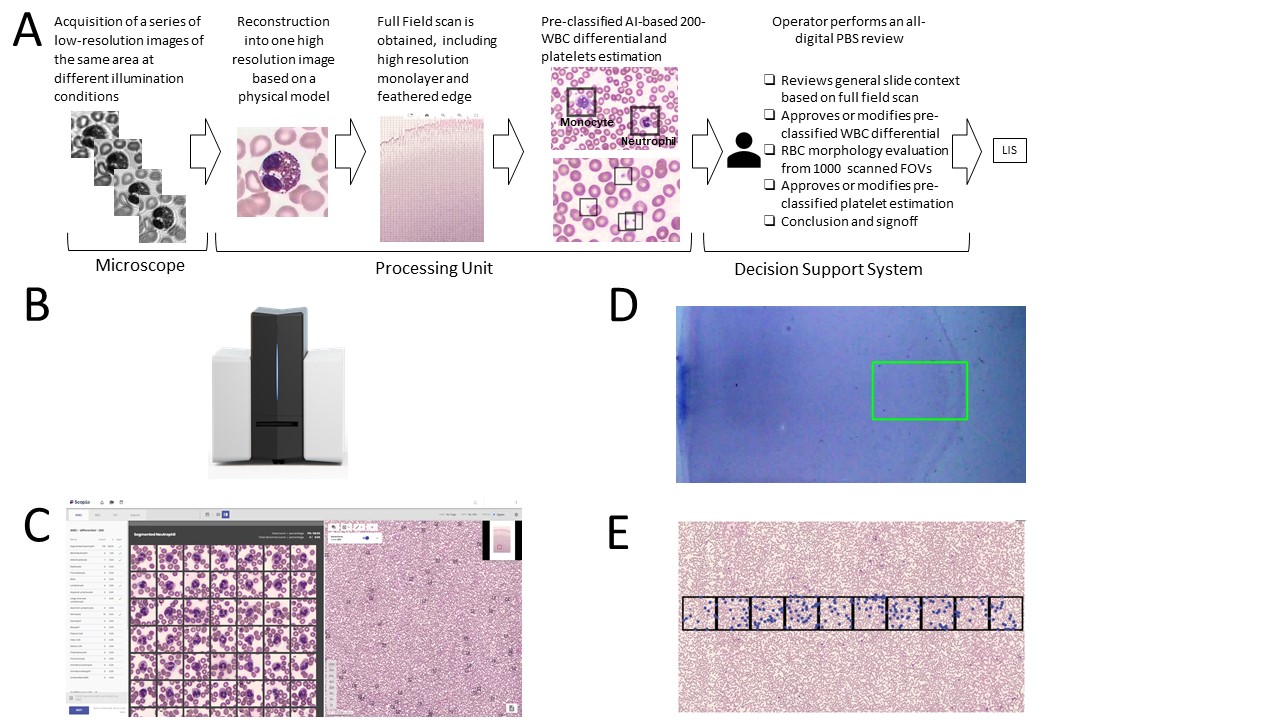
**

**Supplementary 1:** The Scopio Labs X100 system with the Scopio Labs Full Field PBS application. **A**. Scopio Labs X100 mode of operation. Multiple low resolution full field images at various structured illuminations of the specimen are captured by low power/wide field objective. The following processing stages are software driven. The images are reconstructed into a high-resolution full field image based on a physical model. The full field scan includes the monolayer area and the feathered edge. 200 WBC differential and platelets estimation from ten fields are automatically premade by AI-derived tools for operator review. In this Decision Support System (DSS) setting, the operator approves or modifies the WBC differential and platelet estimation. RBC morphology evaluation is done manually based on full field images. The data are summarized in a report, and exported to the Laboratory Information System (LIS). **B**. The Scopio Labs X100 system. **C**. The interactive WBC classification split screen of the Scopio Labs Full Field PBS. Left pane is a thumbnail based view. Right pane is the full field view of the slide. Cells’ thumbnails are easily located within the full field view. **D**. The full field region (green rectangle) in a blood smear (X20 magnification). **E**. The ten fields utilized for platelets concentration estimation (annotated platelets are marked with blue).
