# Supplementary material for: Evaluation of Scopio Labs X100 Full Field PBS: The first high‐resolution full field viewing of peripheral blood specimens combined with artificial intelligence‐based morphological analysis
Source: Int J Lab Hematol. 2021 Sep 21;43(6):1408–16. doi: 10.1111/ijlh.13681 (PMC9293172; doi:10.1111/ijlh.13681)
Supplement: Supplementary file 2 — Supplement S2 [file IJLH-43-1408-s005.docx]

| **Patients characteristics** | |
| --- | --- |
| **Age Group** | **% of total** |
| Neonate (<1 month) | 3.41% |
| Infant (1 month-2 years) | 1.09% |
| Child (3-12) | 0.31% |
| Adolescent (13-21) | 2.79% |
| Adult (22-39) | 19.07% |
| Adult (40-55) | 30.23% |
| Adult (56-74) | 33.33% |
| Adult (>75) | 9.77% |

| **Sex** | **% of total** |
| --- | --- |
| Male | 49.15% |
| Female | 50.85% |

**Supplementary 2**: Demographic characteristics of the 645 individuals that provided the samples for the study.
