# Supplementary material for: Evaluation of Scopio Labs X100 Full Field PBS: The first high‐resolution full field viewing of peripheral blood specimens combined with artificial intelligence‐based morphological analysis
Source: Int J Lab Hematol. 2021 Sep 21;43(6):1408–16. doi: 10.1111/ijlh.13681 (PMC9293172; doi:10.1111/ijlh.13681)
Supplement: Supplementary file 3 — Supplement S3 [file IJLH-43-1408-s007.docx]

**Supplementary 3**

Staining protocols

Site #1: Methanol (Certified ACS, Fisher Chemical, Canada): 1 minute; Hema G (Modified Wright – Giemsa Stain (Fisher Scientific, Pittsburgh PA): 3 minutes (stain ratio: 1:10, stain dilution time: 4 minutes) ColorWright Phosphate Buffer solution pH 6.8, (Sysmex America, Inc Lincolnshire, IL), Cellpack DCL, rinsing agent for spreader glass, sampler pipette, and piercer (Sysmex  America, Inc Lincolnshire, IL); Dry time: 3 minutes. All slides were covered with Surgipath Cover Glass, by using EUKITT® Quick-hardening mounting medium Glue.

Site #2: Methanol (Certified ACS, Fisher Chemical, Canada): 1 minute; 100% Wright Stain (TruColor, Beckman Coulter, Brea, CA, USA): 6 minutes; 5% Wright Stain and 95% Wright Buffer (TruColor, Beckman Coulter, Brea, CA, USA): 6 minutes; D.I. Water: 1 minute; Air dry: 5 minutes. All slides were covered with Fisherbrand Premium Cover Glass, by using Thermo Scientific Richard-Allan Scientific Cytoseal 60 Glue.

Site #3: Methanol (absolute, Bio-Lab Ltd., Jerusalem, Israel): 4 minutes 1 second; May Grunwald Stain (TruColor, Beckman Coulter, Brea, CA, USA): 6 minutes 22 seconds; Water (double distilled, Terion, Cary, NC): 3 minutes; Giemsa Stain (TruColor, Beckman Coulter, Brea, CA, USA): 9 minutes; Water (double distilled, Terion, Cary, NC): 1 minutes; Air dry: 6 minutes. All slides were covered with Süsse Cover Glasses, by using EUKITT® Classic Mounting Medium Glue.
