# Supplementary material for: Evaluation of Scopio Labs X100 Full Field PBS: The first high‐resolution full field viewing of peripheral blood specimens combined with artificial intelligence‐based morphological analysis
Source: Int J Lab Hematol. 2021 Sep 21;43(6):1408–16. doi: 10.1111/ijlh.13681 (PMC9293172; doi:10.1111/ijlh.13681)
Supplement: Supplementary file 4 — Supplement S4 [file IJLH-43-1408-s002.docx]

| **RBC Group** | **Morphological Characteristics** |
| --- | --- |
| Color | Polychromatic  Hypochromatic |
| Size | Anisocytosis  Microcytes  Macrocytes |
| Shape | Poikilocytosis  Target cells  Schistocytes  Helmet cells  Sickle cells  Spherocytes  Elliptocytes  Ovalocytes  Tear drops  Stomatocytes  Acanthocytes  Echinocytes |
| Inclusions | Howell-Jolly bodies  Pappenheimer bodies  Basophilic stippling  Parasites |
| Arrangement | Rouleaux |

**Supplementary 4**. RBC characteristics for comparison between manual microscope and Scopio Labs Full Filed PBS. RBC characteristics were subdivided into 5 groups: color, shape, size, inclusions and arrangement.
