# Supplementary material for: Evaluation of Scopio Labs X100 Full Field PBS: The first high‐resolution full field viewing of peripheral blood specimens combined with artificial intelligence‐based morphological analysis
Source: Int J Lab Hematol. 2021 Sep 21;43(6):1408–16. doi: 10.1111/ijlh.13681 (PMC9293172; doi:10.1111/ijlh.13681)
Supplement: Supplementary file 5 — Supplement S5 [file IJLH-43-1408-s008.docx]

| Cell Type | Arithmetic Mean Differences | 95% LOA | |
| --- | --- | --- | --- |
|  |  | Lower Limit | Upper Limit |
| Neutrophil (%) | 0.26  (-0.07 to 0.58) | -7.62 | 8.13 |
| Lymphocyte (%) | 0.66  (-1.01 to -0.31) | -9.11 | 7.80 |
| Monocyte (%) | -0.54  (-0.71 to -0.36) | -4.64 | 3.57 |
| Eosinophil (%) | -0.35  (-0.48 to -0.22) | -3.49 | 2.78 |

**Supplementary 5**. Bland-Altman analysis of the test and reference methods for neutrophils, lymphocytes, monocytes and eosinophils (in %). The arithmetic mean differences and the 95% limits of agreement (LOA) for each parameter are indicated.
