# Supplementary material for: Evaluation of Scopio Labs X100 Full Field PBS: The first high‐resolution full field viewing of peripheral blood specimens combined with artificial intelligence‐based morphological analysis
Source: Int J Lab Hematol. 2021 Sep 21;43(6):1408–16. doi: 10.1111/ijlh.13681 (PMC9293172; doi:10.1111/ijlh.13681)
Supplement: Supplementary file 6 — Supplement S6 [file IJLH-43-1408-s004.docx]

|  | | Scopio Labs X100 pre-classified *vs* Manual | Scopio Labs X100 with user re-classification *vs* Manual |
| --- | --- | --- | --- |
| WBC | Efficiency % | 92.99 | 96.29 |
|  | Sensitivity % | 80.78 | 87.86 |
|  | Specificity % | 94.92 | 97.62 |
| Platelets | Efficiency % | 94.21 | 94.89 |
|  | Sensitivity % | 89.23 | 90.00 |
|  | Specificity % | 95.62 | 96.28 |

**Supplementary 6.** Re-classification impact on performance

- Efficiency | Overall agreement: quantifies the percentage of overall cases agreement between the two methods (manual differential count vs Scopio Labs Full Field PBS).
- Sensitivity | Abnormality agreement: quantifies the percentage of clinical abnormal cases agreement between the two methods (manual differential count vs Scopio Labs Full Field PBS).
- Specificity | Normality agreement: quantifies the percentage of clinical normal cases agreement between the two methods (manual differential count vs Scopio Labs Full Field PBS).
- Regarding blast cells, system pre-classification showed efficiency of 97.93%, sensitivity of 82.61% and specificity of 99.25%, which were improved to 99.48%, 97.83% and 99.62% by manual reclassification.
