# Supplementary material for: Evaluation of Scopio Labs X100 Full Field PBS: The first high‐resolution full field viewing of peripheral blood specimens combined with artificial intelligence‐based morphological analysis
Source: Int J Lab Hematol. 2021 Sep 21;43(6):1408–16. doi: 10.1111/ijlh.13681 (PMC9293172; doi:10.1111/ijlh.13681)
Supplement: Supplementary file 7 — Supplement S7 [file IJLH-43-1408-s003.docx]

| Cell Type | Arithmetic Mean Differences | 95% LOA | |
| --- | --- | --- | --- |
|  |  | Lower Limit | Upper Limit |
| Platelets Estimation (10^3^/µL) | -3.40  (-6.59 to -0.21) | -80.52 | 73.72 |

**Supplementary 7**. Bland-Altman analysis of the test and reference methods for platelets. The arithmetic mean differences and the 95% limits of agreement (LOA) for each parameter are indicated.
