# Supplementary material for: Evaluation of Scopio Labs X100 Full Field PBS: The first high‐resolution full field viewing of peripheral blood specimens combined with artificial intelligence‐based morphological analysis
Source: Int J Lab Hematol. 2021 Sep 21;43(6):1408–16. doi: 10.1111/ijlh.13681 (PMC9293172; doi:10.1111/ijlh.13681)
Supplement: Supplementary file 8 — Supplement S8 [file IJLH-43-1408-s006.docx]

| Designation | Morphological feature | Link |
| --- | --- | --- |
| A | Schistocytes | <https://demo.scopiolabs.com/#/view_scan/4c39d956-d83d-4708-9b40-4cc4bc263046> |
| B | Malaria | <https://demo.scopiolabs.com/#/view_scan/e53834f9-9e69-4d65-affe-a403837a894b> |
| C | Rouleaux | https://demo.scopiolabs.com/#/view_scan/56bd9d98-43bc-409c-91ed-03ea0f1b56b6 |
| D | Platelets clumps | <https://demo.scopiolabs.com/#/view_scan/ade3351f-7e80-48ae-a5be-54ecbb6a7776> |
| E | Platelets clumps | <https://demo.scopiolabs.com/#/view_scan/8b1ce830-e71d-4f82-bfc2-92e397eb91b8> |

**Supplementary 8**. Representation of full filed PBS of RBC and platelets abnormalities
